# Correction: Kusayanagi et al. A Smartphone Application for Personalized Tooth Shade Determination. *Diagnostics* 2023, *13*, 1969

**DOI:** 10.3390/diagnostics13132288

**Published:** 2023-07-06

**Authors:** Tomoya Kusayanagi, Sota Maegawa, Shuya Terauchi, Wataru Hashimoto, Shohei Kaneda

**Affiliations:** Mechanical Engineering Program, Graduate School of Engineering, Kogakuin University, 1-24-2 Nishishinjuku, Shinjuku-ku, Tokyo 163-8677, Japan

In the original publication [1], there was a mistake in Figure 5 as published. The coordinate annotation of (d) in Figure 5 is incorrect. The corrected version of Figure 5 appears below. The authors state that the scientific conclusions are unaffected. This correction was approved by the Academic Editor. The original publication was also updated.

## Figures and Tables

**Figure 5:**
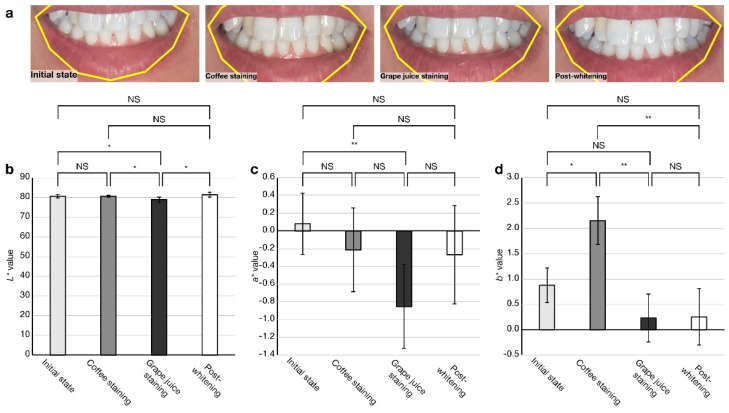
Feasibility check of the app for tooth shade determination. (**a**) Representative images of pre- and post-gel whitening treatment with intermediate pseudo-staining. Color measurement results of (**b**) L* value, (**c**) a* value and (**d**) b* value. (NS, not significant; * *p* < 0.05, ** *p* < 0.01; *n* = 5). Error bars indicate ± SD.
