# Supplementary material for: A Smartphone Application for Personalized Tooth Shade Determination
Source: Diagnostics (Basel). 2023 Jun 5;13(11):1969. doi: 10.3390/diagnostics13111969 (PMC10252511; doi:10.3390/diagnostics13111969)
Supplement: Supplementary file 1 [file diagnostics-13-01969-s001.zip › diagnostics-2384000-Supplementary.pdf]

Supplementary Materials

A Smartphone Application for Personalized Tooth Shade Determination

Tomoya Kusayanagi [1,†], Sota Maegawa [1], Shuya Terauchi [1], Wataru Hashimoto [1] and Shohei Kaneda [1,†,*]

[1] Mechanical Engineering Program, Graduate School of Engineering, Kogakuin University, 1-24-2 Nishi-Shinjuku, Shinjuku-ku, 163-8677 Tokyo;

[†] These authors contributed equally to this work.
[*] Correspondence: kaneda@cc.kogakuin.ac.jp (S.K.);

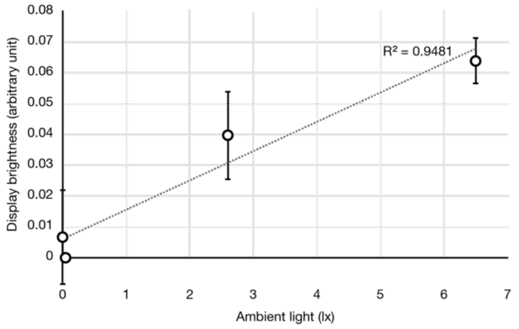

Figure S1. Correlation between the display brightness and ambient light. Error bars indicate SD. $n = 5$.

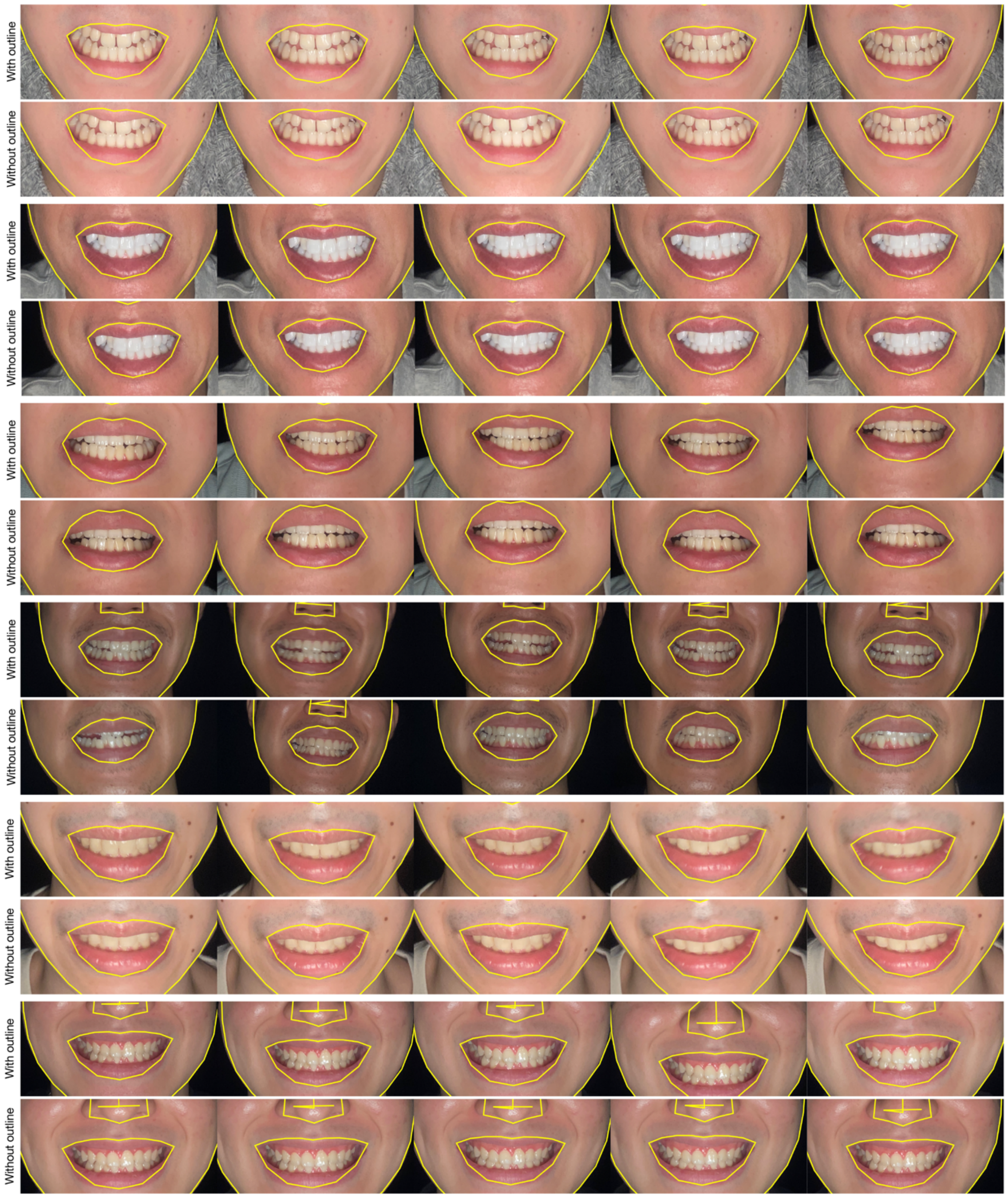

Figure S2. Cropped images of mouth from dental photographs of the six participants.

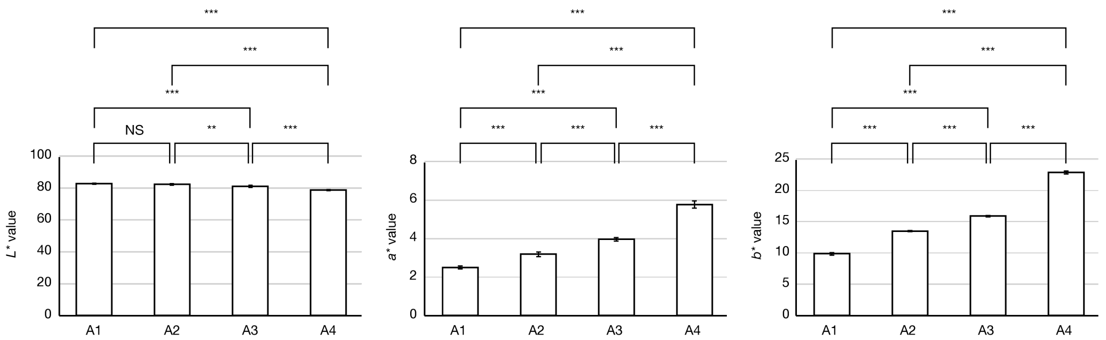

Figure S3. Color measurements of the shade tabs at fixed camera tab distance of 20 cm under 0 lx lighting. (Left) L^* value, (Middle) a^* value, (Right) b^* value. NS, not significant; *, $P < 0.05$, **, $P < 0.01$, ***, $P < 0.001$; $n = 5$.

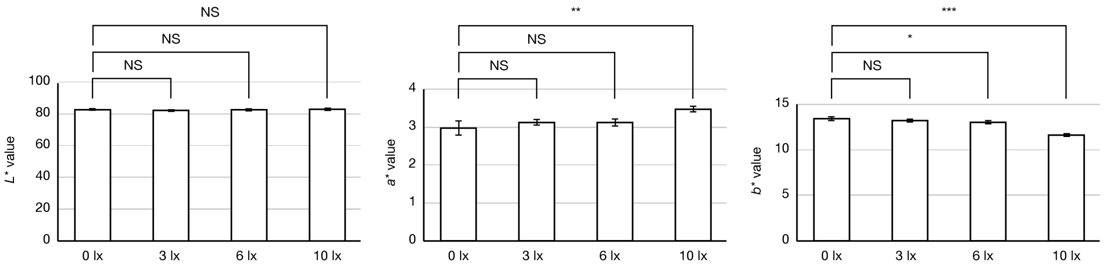

Figure S4. Color measurements of the A2 tabs at fixed camera tab distance of 20 cm under various illumination conditions. (Left) L^* value, (Middle) a^* value, (Right) b^* value. NS, not significant; *, $P < 0.05$, **, $P < 0.01$, ***, $P < 0.001$; $n = 5$.

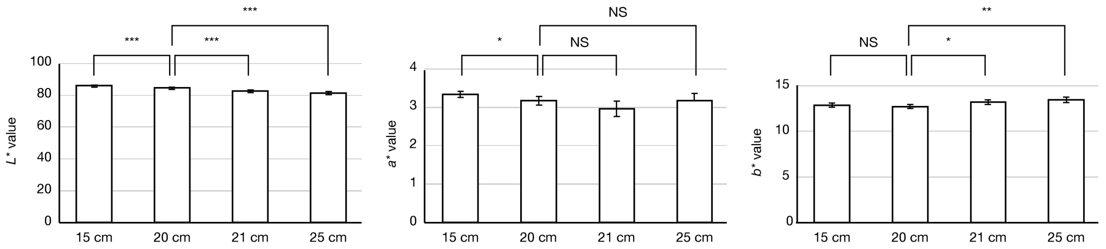

Figure S5. Color measurements of the A2 tabs at various camera tab distance under 0 lx lighting. (Left) L^* value, (Middle) a^* value, (Right) b^* value. NS, not significant; *, $P < 0.05$, **, $P < 0.01$, ***, $P < 0.001$; $n = 5$.

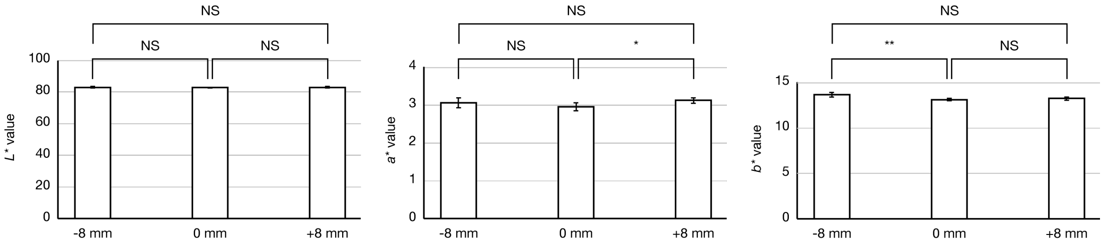

Figure S6. Color measurements of the A2 tabs at fixed camera tab distance of 20 cm with ± 8 mm-horizontal displacement under 0 lx lighting. (Left) L^* value, (Middle) a^* value, (Right) b^* value. NS, not significant; *, $P < 0.05$, **, $P < 0.01$, ***, $P < 0.001$; $n = 5$.

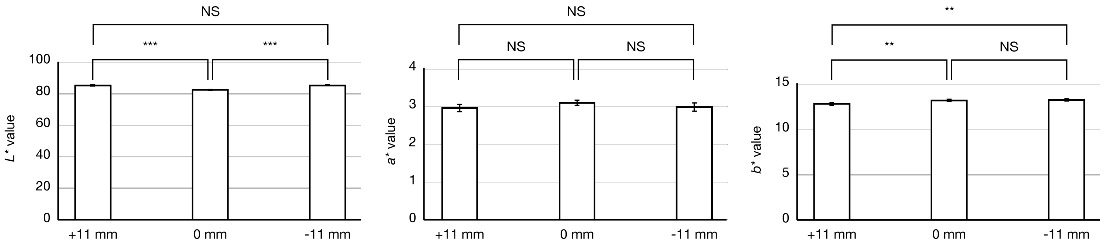

Figure S7. Color measurements of the A2 tabs at fixed camera tab distance of 20 cm with ± 11 mm-vertical displacement under 0 lx lighting. (Left) L^* value, (Middle) a^* value, (Right) b^* value. NS, not significant; *, $P < 0.05$, **, $P < 0.01$, ***, $P < 0.001$; $n = 5$.

Table S1. Color difference between shade tabs obtained at fixed camera tab distance of 20 cm under 0 lx lighting.

ΔE_{ab}	A1	A2	A3	A4
A1	–	3.7	6.5	14.0
A2	3.7	–	2.8	10.3
A3	6.5	2.8	–	7.5
A4	14.0	10.3	7.5	–

Video S1: The movie demonstrates how to capture dental photographs.
Video S2: The movie shows a failure to activate the camera due to insufficient darkness in the ambient light.
Video S3: The movie shows a failure to save photographs due to a lack of uniformity in tooth appearance for tooth position.
Video S4: The movie shows a failure to save photographs due to a lack of uniformity in tooth appearance for tooth area.
